# The effect of habitat changes along the urbanization gradient for breeding birds: an example from the Xiong’an New Area

**DOI:** 10.7717/peerj.7961

**Published:** 2019-10-30

**Authors:** Shilin Xie, Yuebo Su, Weihua Xu, Wenbo Cai, Xiaoke Wang, Fei Lu, Zhiyun Ouyang

**Affiliations:** 1 State Key Laboratory of Urban and Regional Ecology, Research Center for Eco-environmental Science, Chinese Academy of Sciences, Beijing, People’s Republic of China; 2 University of Chinese Academy of Sciences, Beijing, People’s Republic of China; 3 Beijing Urban Ecosystem Research Station, Research Center for Eco-environmental Science, Chinese Academy of Sciences, Beijing, People’s Republic of China

**Keywords:** Xiong’an new area, Urbanization gradient, Micro polis, α  and β-diversity, Avian community

## Abstract

**Background:**

Because of its status as an ecocivilization pilot city, fundamental research on spatial distribution patterns and impact factors of the avian community within the Xiong’an New Area is necessary for future ecological planning and mitigation of negative impacts from future urbanization. Gradient research within small cities can provide important information for the development of urbanization gradient patterns of avian communities.

**Methods:**

A total of 30 sample points within the urbanization gradient were selected, and avian communities and environmental variables were measured within a 50 m radius sample circle. Principal component regression analysis was used to analyze bird-environment relationships. The Sorensen dissimilarity index was used to calculate the beta-diversity.

**Results:**

Our results showed that there was a significant urban-rural pattern with a gradient phenomenon in avian communities. Results of this study showed more resident, passenger and insectivore species, and a higher density of breeding insectivore and omnivore individuals appear in the urban fringe than in the other areas. A relatively high value of overall beta-diversity and spatial isolation probably exists among the three disjunct constructed regions. Both species richness and individual abundance were significantly influenced by the species diversity of the trees and foliage height diversity (FHD).

**Discussion:**

Based on our results and our goal of avian species diversity conservation, we first suggest that urban green spaces be established and ensure a high complexity of vegetation structure as this is critically needed to increase avian species *α*-diversity within habitat patches. Second, different habitat types within and around the three constructed areas should be developed based on the important existing bird habitats to increase avian diversity in each city, especially in the areas within Xiongxian and Rongcheng that are well protected, and to elevate the beta-diversity of the total region. Finally, based on the biodiversity hotspots identified by this research, ecological corridors should be carefully planned to improve the stability of regional bird communities.

## Introduction

The global urban population will likely reach 5 billion by 2025, and China, as the largest developing country, will contribute to a large part of this total (DESA, 2007). Urbanization will generate intensively constructed urban centers and moderately renovated urban fringes (Mcdonnell & Pickett, 1990), but urbanization is a major threat to global biodiversity (Sala et al., 2000), often accompanied by irreversible damage to natural ecosystems (Seto, Güneralp & Hutyra, 2012). As some of the top organisms in the food chain, birds have long been used to monitor environmental change and its related effects (Chace & Walsh, 2006; Marzluff, 2016). As important indicators of biodiversity conservation and human environments, avian species richness and individual abundance are representative of the value of not only urban green spaces (Magle et al., 2012; Shwartz et al., 2014) but also urban ecosystem functions and services (Lin & Fuller, 2013; Mainwaring, 2017; Yam et al., 2015).

To date, a myriad of studies has focused on urbanization and its impact on avian communities (Jokimäki et al., 2015; Marzluff, 2001). Beta-diversity between cities has exhibited a general decrease (Aronson et al., 2014) as a result of a decrease in the distance decay of compositional similarities between cities due to urbanization (Ferenc et al., 2014). However, beta-diversity within specific cities could be elevated by species dispersal limitations and neutral processes originating from habitat fragmentation (Püttker et al., 2015). In comparison to beta-diversity, distribution patterns and influencing factors of avian communities within fragmented habitat patches are more complicated. With the removal of original natural land cover, urbanization drives decline in species, functional and phylogenetic diversity and also the increasement of functional redundancy (Palacio et al., 2018). Avian distribution patterns along an urbanization gradient most likely depend on the development type and surrounding environmental matrix of the urban region being studied. For example, Marzluff (2001) suggested that avian species richness undergoes a continued decrease corresponding to an increase in urbanization degree, and individual density and biomass act in the opposite manner. While recent studies suggested that the urbanization gradient pattern differed between species guilds (Kale et al., 2018), continents (Clucas & Marzluff, 2015) and will change by time (Palacio et al., 2018). In regard to breeding bird individual density, a consistent decrease occurs (Tratalos et al., 2007). Urbanization could exert large influences on breeding bird populations through the destroy of nest sites and the eliminating of food resources (Han et al., 2019; Hensley et al., 2019). Additional gradient studies have revealed that along an urbanization gradient, avian species richness peaks in moderately urbanized areas (Blair, 1999; Jokimaki & Suhonen, 1993; Pal et al., 2019; Verma & Das Murmu, 2015), which is the intermediate disturbance hypothesis (Connell, 1978), while a recent study suggested that it depends on the end of the gradient and biome (Filloy, Andres Zurita & Isabel Bellocq, 2019). Studies focusing on the avian-environment relationship have generally shown that in comparison to regional factors, local habitat configuration has a greater effect on this relationship (Evans, Newson & Gaston, 2009), especially for species with limited dispersion capacity (Graham & Blake, 2001). For example, most bird species positively respond to habitat area (Carbo-Ramirez & Zuria, 2011; Chaiyarat et al., 2019; Lepczyk et al., 2017), though the response may decrease when patch area reaches a threshold (Kim, Chae & Koo, 2007). Increased coverage of trees (Rega-Brodsky & Nilon, 2017), shrubs (Munoz-Pedreros et al., 2018), and vegetation structure heterogeneity due to different tree ages and large or multiple canopy layers (Franklin & Van, 2004; Lindenmayer, Franklin & Fischer, 2006) seem to be crucial for avian species richness (Chong et al., 2014; Lee & Rotenberry, 2015). The effect of shrubby and herbaceous species is probably exerted through the insect communities they sustain (Huang et al., 2015), which is consistent with the results of studies that have emphasized the importance of vegetative food resources (Chace & Walsh, 2006). On the other hand, anthropogenic noise and pedestrians are the major kinds of human disturbance that are typically negatively correlated with avian communities (Antonio Gonzalez-Oreja, 2017; Perillo et al., 2017; Zhou & Chu, 2012), which is the premise of the hypothesis of flush distance research (Levey et al., 2009). The building cover was suggested to be important for several functional groups, which will change over time (Palacio et al., 2018). However, existing urbanization gradients and avian-environment research have mostly focused on metropolitan areas with land cover that has been intensively transformed due to urbanization (Crooks, Suarez & Bolger, 2004; Sengupta, Mondal & Basu, 2014; Shuping, 2008), and small cities such as some in Chinese counties have seldomly been included. A small county is at the threshold of being a city and could be the origin of a city. Avian community distribution patterns within small cities could supply crucial information about the evolution of a gradient distribution of urban birds. Although one related study has suggested that there is a threshold for urbanized areas, the influences of urbanization on urban birds above a threshold can be significantly exaggerated (Garaffa, Filloy & Bellocq, 2009). Under the background of consistent increasing of urbanization pressure within our study area, the effort to find out the efficient methods to control its damage on avian communities becomes highly important.

On April 1, 2017, the Chinese government officially announced the establishment of the Xiong’an New Area, which includes Xiongxian (XX), Anxin County (AX) and Rongcheng County (RC) of Baoding, Hebei Province. The special area will be an important node of the Jing-Jin-Ji (Beijing-Tianjin-Hebei) urban cluster, and the original intention of the Chinese government was to relieve the noncapital function of Beijing and to optimize the urban structure of the Jing-Jin-Ji region. Based on the traffic and environmental problems from previous urban construction and development, policymakers made ecological and green development the top priority in the construction of the Xiong’an New Area, resulting in a law requiring the establishment of an ecoenvironmental model ecocivilization. While biodiversity conservation is one of the most important parts of ecocivilization construction, descriptions of community structure and spatial distribution patterns and the differentiation between key environmental factors of avian communities based on field surveys and statistical analysis could provide critical information for ongoing ecoenvironmental construction and further research.

Thus, based on previous research, this study focused on the following factors: (1) avian community structural characteristics of small cities on the North China Plain during the breeding season, whether their spatial distribution patterns along the urbanization gradient were consistent with those in other studies that focused on metropolitan areas? We hypothesize that both avian species richness and individual abundance peak at the urban fringe, as green space is largely deficient in the urban region and rural area; (2) major environmental factors for those avian communities, we suggest that the plant configuration and the related food resources will be dominant in the decision of avian communities, while interference factors like environment noise and pedestrian number will be less important because of the trade-off between survival and adventure; (3) and on the basis of the first two points, whether the avian biodiversity hotspots within the study area further provide ecological information to assist in the construction of ecological corridors.

## Methods

### Study sites

**Figure 1:**
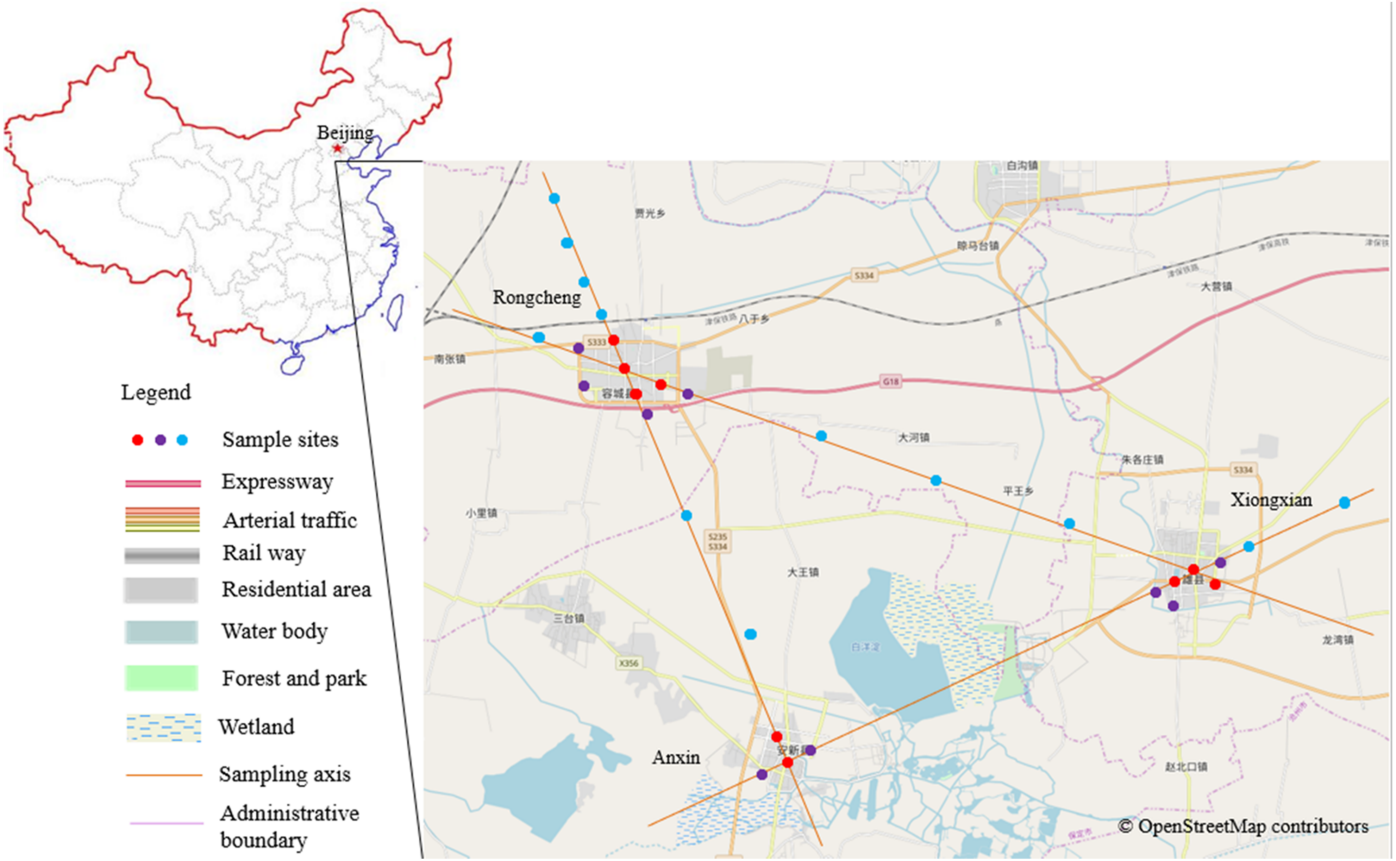
Research district and the distribution map of the sampled plots. Different colors of the sample points represent different urbanization gradients, that is, red for urban plots, purple for urban fringe plots, and blue for rural plots. The base map is OpenStreetMap ©OpenStreetMap contributors (URL: http://www.openstreetmap.org/export#map=12/39.8602/116.3507&layers= H), the cartography in the OpenStreetMap map tiles is licensed under CC-BY 4.0 license (http://www.openstreetmap.org/copyright). And the map was processed by ArcGIS 10.2 (URL: http://www.esri.com/).

The total size of the Xiong’an New Area is 1,566 km^2^ (38°43′–39°10′N, 115°38′–116°20′E). The elevation of our study area ranges from 7 m to 19 m. Historically, this area was part of the Central Plains region of ancient China, and the landscape has experienced intensive agricultural exploitation for more than 2,000 years. In addition to land being used for construction, the other main land-use types are agricultural, with the main crop being wheat (*Triticum aestivum L.*), and rows of poplar (*Populus X canadensis Moench*) planted at the boundary of some farmland that act as shelterbelts. The total vegetation coverage is 63.75% (Xu et al., 2017), and Baiyangdian, the largest lake within Hebei Province, is located between AX and XX (Fig. 1). A recent survey showed that there were 72 bird species (among which 51 were forest birds and 21 were water birds) that occurred in the summer and winter seasons in this area (Zhou et al., 2018). The area has a warm temperate continental monsoon climate with distinctive seasons and an average temperature of 12.1 °C. By the end of 2015, there were 1.13 million permanent residents within the area, and the urbanization rate was 42.74% (Baoding Statistical Bureau, 2016).

### Field surveys

Based on the relative spatial position and configuration of the three constructed areas of the Xiong’an New Area (Fig. 1), we first determined the geometric center points of the three cities and connected each pair of the three points with three straight lines. Sample points were then selected along the lines originating from the three city centers, and representative sample points were selected every 1–4 km outward from the three centers (Bibby, Jones & Marsden, 1998) that is, 1 km for urban and urban fringe points to ensure the independency of each field count, 1 or 4 km for rural points, we didn’t chose rural points by 1 km separation for all of the survey because of the unanimous configuration of the crisscross rural fields. Within the three constructed areas, densely constructed buildings and roads dominate the landscape, and each area is surrounded by an obvious edge that extends between the constructed area and agricultural land. Given these landscape patterns, we classified our sampled points into a specific urbanization gradient based on landscape construction and spatial position; that is, points that were surrounded by more than 50% of artificial land cover within a 200 m radius buffer region and that were located inside the city edge were defined as the urban points, points that were surrounded by 20–50% artificial land cover and located within 100 meter from the city edge were urban fringe points, and points with artificial land cover totaling less than 20% and agriculture land cover totaling more than 50% within the 200 m buffer region were classified as rural points (Verma & Das Murmu, 2015). To ensure spatial independence, the minimum distance between each pair of sample points was 1 km (Legendre et al., 2002). A total of 30 sample points were selected (Fig. 1 and Table 1).

**Table 1:** Environmental factors and breeding bird community characteristics of this research with means and ranges.

**Sample Type**	**Number of samples**	**Den(ha)**^a^	**Spe**^b^	**S-arbor**	**C**^c^**-arbor**	**C-herb**	**Distance (Km)**^d^	**FHD**^e^
Rural	12	18.42(5.10–71.34)	2.75(1.00–6.50)	0.83(0–3.00)	15%(0%–95%)	77%(2%–100%)	5.60(1.76–10.50)	3.20(2.00-4.43)
Urban Fringe	9	56.69(14.65–128.66)	7.44(3.50–10.00)	9.00(3.00–21.00)	65%(23%–90%)	55%(1%–90%)	1.96(1.15–2.77)	4.09(3.14–5.57)
Urban	9	27.32(8.92–54.78)	3.28(1.00–7.00)	8.44(3.00–23.00)	21%(3%–46%)	22%(1%–97%)	0.72(0–1.12)	3.15(2.42-4.11)

**Notes.**

a Breeding bird individual density.

b Breeding bird species richness.

c Coverage.

d The shortest distance between sample points and the nearest urban region.

e Foliage height diversity.

### Bird survey and anthropogenic disturbance records

The fixed-point count method (Bibby et al., 2000) was adopted for our bird survey in the breeding season (April 2017 and June 2018), and every point was sampled at least two times. The field survey was conducted only on days with good weather (no rain or strong winds with speeds of more than 30 km/h), from 6:30∼10:30 and 16:00∼18:00 on each day when birds were active and thus easy to detect. Bird census began at the time the surveyor arrived at the center of the sample point, and bird species and individual birds heard or seen within a 50 m radius were recorded for 5 min. Individuals flying through the sample points that did not stop were excluded from our count. As a typical representation of anthropogenic disturbance, the number of pedestrians within the bird census circles during the bird count period was recorded, and environmental noise was measured just after the bird census finished. Environmental noise level was measured with a high precision noise meter (SW-524) in four directions, 25 m from the center of each sample point. The highest and lowest values within 20 s were recorded for each measurement, and the average value of the census within each sample point was used as its environmental noise level. To reduce the survey error among different investigators, all of the surveys of the birds in this study were completed by one investigator (the first author of this paper, with six years of experience of bird watching and field surveys). The field survey was approved by the Research Council of Research Center for Eco-environmental Science, Chinese Academy of Sciences.

### Vegetation survey

Vegetation surveys were conducted at the same time as the bird count, and plant species (including arbor, shrub and herb) and the individual number of trees (more than 1-meter height) were recorded within the 50 m radius circle. Multiple layers of coverage were evaluated based on visual estimation by an experienced plant surveyor. The vertical structure of the vegetation was measured as the foliage height diversity (FHD). From the center of each sample point, four observing line (50 m long) which are perpendicular to each other were decided with a north arrow, observation points were chosen at 10-m intervals along each line. The presence of leaves at different vertical height levels (0–1, 1–2, 2–5, 5–10, 10–20, and 20–30 m) was observed and recorded at each point, using a 5-m-long pole as visual reference. For details, please see Lee & Rotenberry (2015).

### Data analysis

Avian species richness (Spe) was measured as the number of species recorded at each sample point. Tree species diversity and FHD were calculated using the Shannon-Wiener index, and the formula is H′ = ∑PilogPi, P_i_ is the ratio of the individual number of species I and total individual number. The individual density (Den) of each urbanization gradient was calculated using $ \frac{\mathrm{Den}=\sum {\mathrm{N}}_{k}}{\pi {\mathrm{r}}^{2}} $ , where N is the individual number of sample point k, and r is the radius of the sample point (50 m).

Based on resident types, all birds recorded in the field survey were classified as residents, passengers, summer visitors or winter visitors (Zheng, 2017). Based on feeding types, all birds were defined as insectivores (I), granivores (G), insectivore-frugivores (IF), omnivores (O) or carnivores (C) (Kwok & Corlett, 2000). The three city areas of the Xiong’an New Area were spatially independent, and the minimum distance between each pair of cities exceeded 10 km, separated by agricultural land and villages. A comparative analysis for avian communities between independently constructed areas could provide critical information for the master plan of the newly established special zone. To compare avian community structure between cities and urbanization gradients, we defined bird species as exclusive species when they only appeared in a single city.

To compare the differences between bird guilds of each urbanization gradient and constructed area, one-way ANOVA was used to check whether the differences between avian species richness and individual abundance within each gradient or city had reached significant levels. To further understand overall bird species diversity within the study area and to describe the ecological driver of current species distributions, beta-diversity of avian community between city pairs was calculated as the multisite Sorensen dissimilarity index (β_SOR_). To understand the sources of species compositional differences, β_SOR_ was divided into two additive components, β_NES_(nestedness) and β_SIM_(turnover) (Baselga, 2010), and the ratio between them (β_NES_/β_SIM_) represented the relative contribution of the two components (Quan et al., 2018). Considering only the overall diversity and ignoring its respective components may lead to deviations in the formulation of conservation strategies (Angeler, 2013). Thus, pairwise dissimilarity and the corresponding additive components (βsor, βsim, βnes, and βsim/βnes) were also calculated. In this study, only breeding bird species were utilized in beta-diversity calculation and the subsequent avian-environment correlation analysis, which could help avoid errors resulting from the occasional occurrence of passenger birds.

Unfortunately, only 25% of the total sample points contained shrub layers, and thus, shrub indexes were excluded from further analysis. In our further analysis, pedestrian number, environment noise, tree species richness, diversity and coverage of trees, and FHD are predictors, which have all been demonstrated to be important for urban birds in other studies, and avian species richness and individual abundance are the response variable. To ensure the normality of the data, all variables were log-transformed and conformed to a normal distribution. Multicollinearity impairs the reliability of regression analysis (Mac Nally, 2000), and to resolve this problem, we first utilized Spearman’s correlation to determine the environmental factors that showed significant correlation with avian community indexes. Then, we used principal component analysis (PCA) to extract the principle components, and further, stepwise regression was conducted, with the principal component scores being the predictors and avian species richness and individual abundance are the response variables. Beta-diversity was calculated using the “betapart” package of R 3.5.1 (Baselga & Cdl, 2012), and other analyses were conducted using SPSS 22.0.

## Results

### Avian community characteristics recorded in the field survey

**Figure 2:**
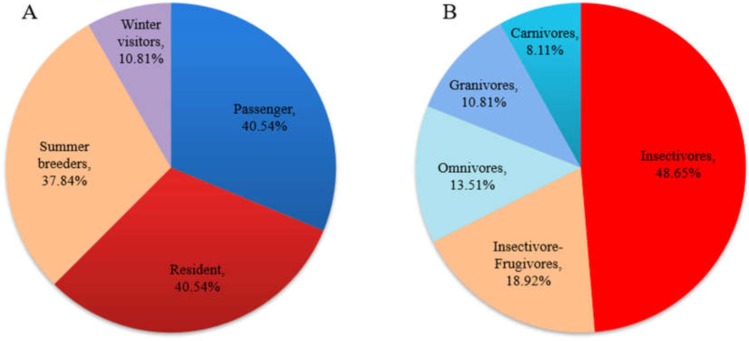
Residence (A) and feeding-type (B) structure of birds recorded in the field survey.

A total of 37 bird species (from nine orders and 26 families) were recorded in the field survey. For resident types, residents and passengers accounted for the majority of these species and accounted for the same proportion (40.54%); breeding birds (including residents and summer visitors) accounted for 78.38% of the total species number. In terms of feeding type, insectivores accounted for the largest proportion of the birds (48.65%), followed by insectivore-frugivores (18.92%) (Fig. 2). Three national class II protected bird species (all raptors) were recorded. In this study, taxonomy and residence type were based on *A Checklist on the Classification and Distribution of the Birds of China (Third Edition)* (Zheng, 2017). The species list and the supplemental information are provided in Appendix S1.

### Community characteristics within different urbanization gradients

**Figure 3:**
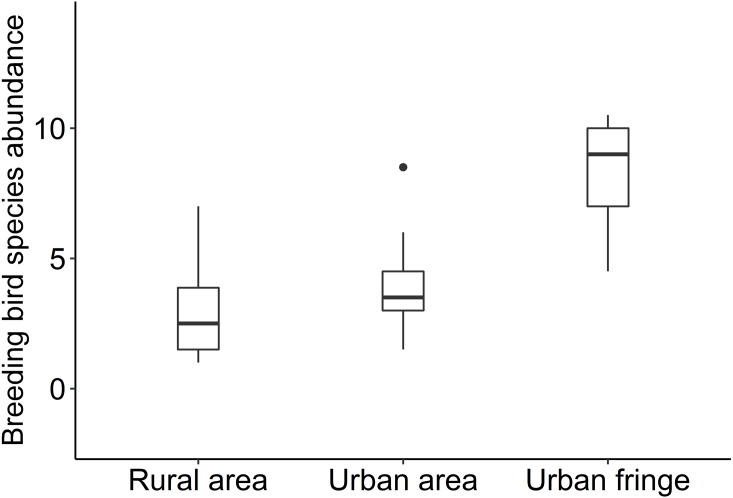
Box plots of breeding bird species abundance for the urbanization gradients.

**Figure 4:**
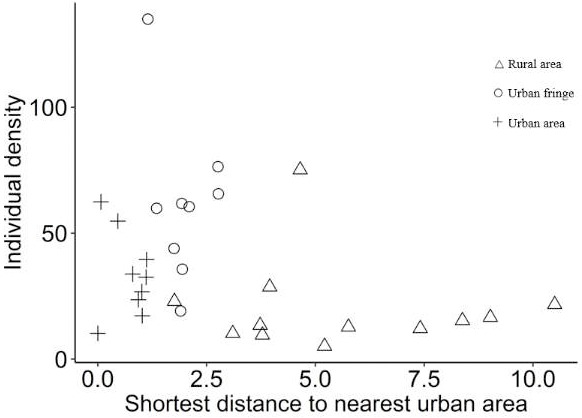
Scatter plots of breeding bird individual density (/ha) for the urbanization gradients.

**Figure 5:**
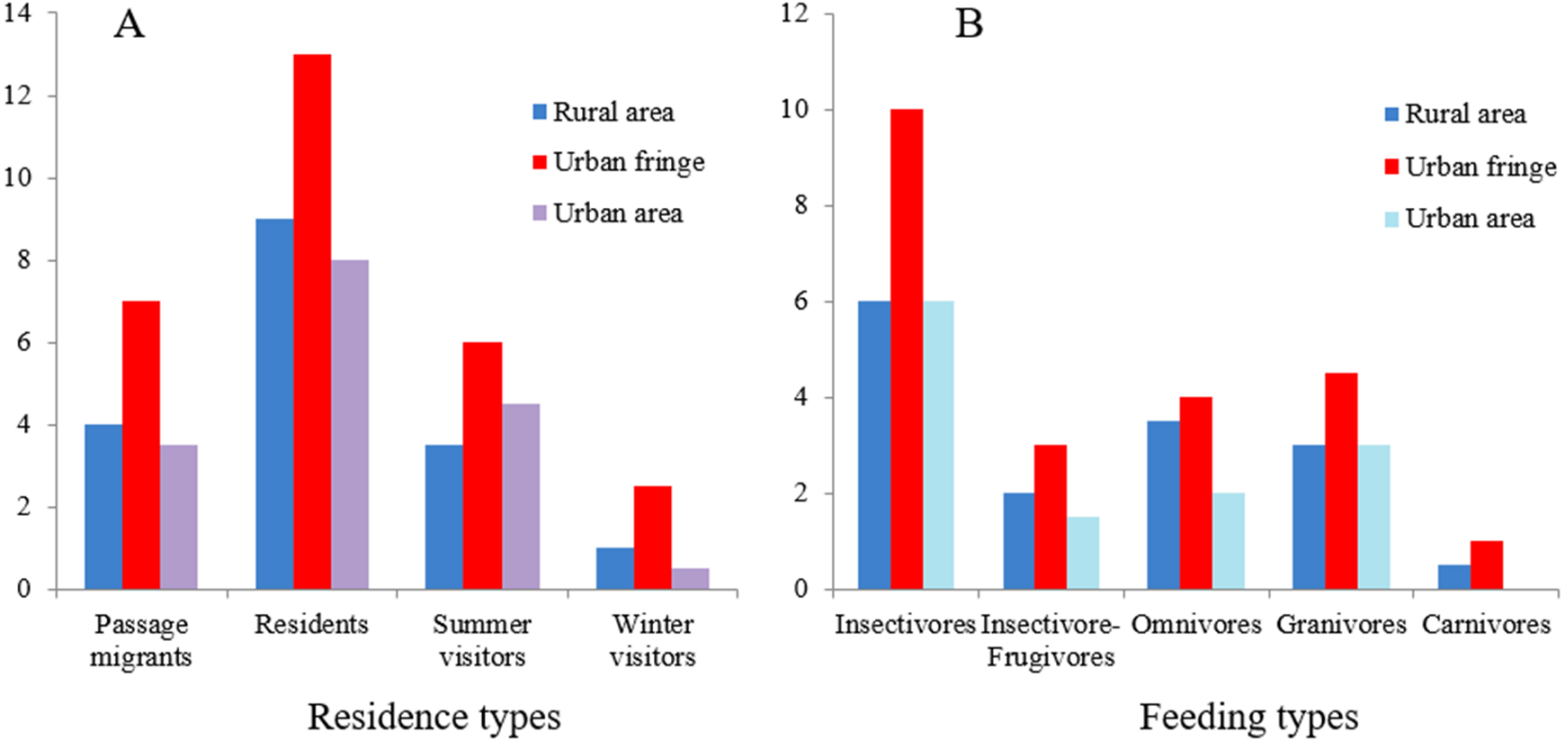
Residence (A) and feeding-type (B) structure of total birds at different urbanization levels.

**Figure 6:**
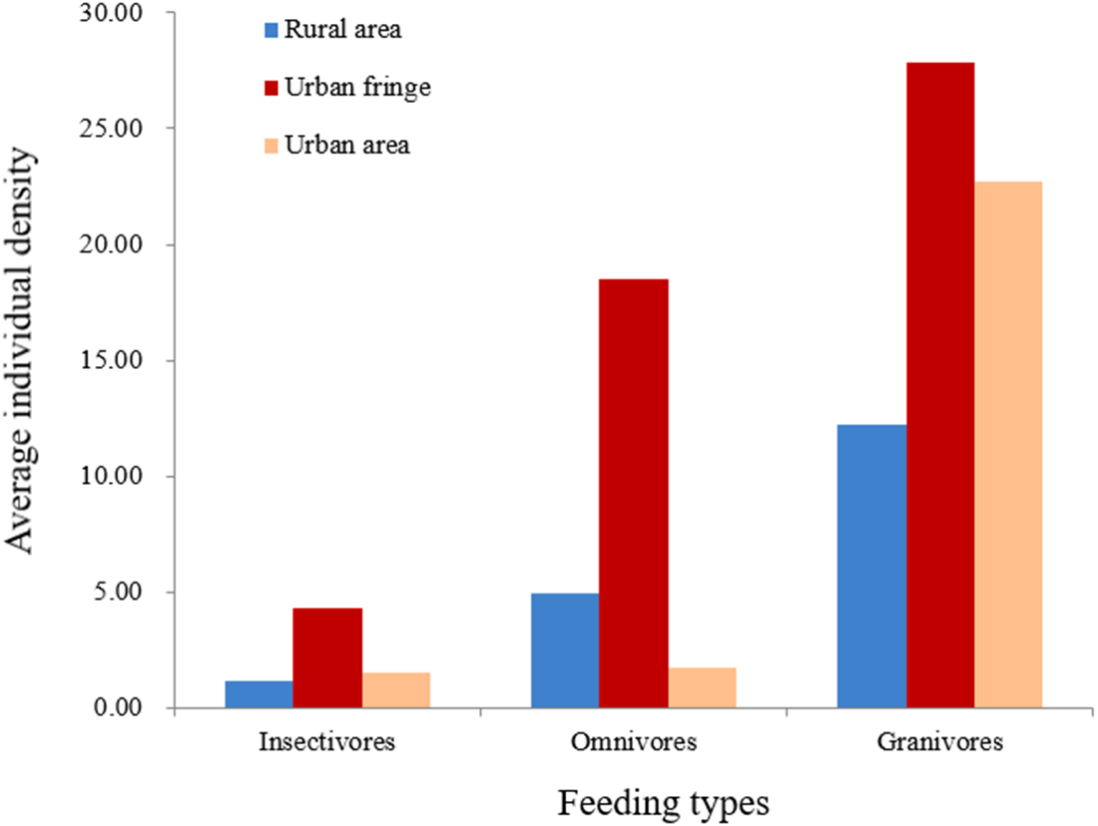
Breeding bird average individual density (/ha) for different feeding types.

Significant differences between species richness (*F* = 13.42, *P* < 0.001) and individual abundance (*F* = 10.94, *P* < 0.001) for the three urbanization gradients were detected by one-way ANOVA (Figs. 3 and 4). A total of 22 bird species were recorded in the rural area, three of which were exclusive species (including two breeding birds); 34 bird species were detected within the urban fringe, with eight exclusive species (including five breeding birds); only 18 bird species were found within the urban area, with two exclusive species (only one breeding bird). Nine occurred in all urbanization gradients, and all nine were breeding birds; 15 species were shared by two of the three gradients, of which 12 were breeding birds; and 13 species existed within only one gradient, of which eight were breeding birds (Table 2). Different guilds of bird species showed similar distribution patterns in the urbanization gradient (urban fringes contained all peaks), but the pattern was more obvious for species richness of residents, passengers, insectivores (Fig. 5), and omnivore individual density (Fig. 6).

**Table 2:** Species distribution characteristics among the three counties.

	Total species number	Exclusive species number	Breeding exclusive species number
Rural area	22	3	2
Urban fringe	34	8	5
Urban region	18	2	1
	Total species number		Breeding species
Species shared by all cities	9		9
Species shared by two cities	15		12
Species occurred in on single city	13		8

### Avian community characteristics of the three constructed areas

Statistical analysis results showed that 13 of the 37 bird species (37.14%) were exclusive species of one specific city; six species (17.14%) were shared by two cities, and only 16 species (45.71%) were shared by all cities. The results for breeding bird species are as follows: eight species (27.59%) were specific to one city, five species (17.24%) were specific to two cities, and 16 species (55.17%) were shared by all species. The difference among the breeding bird species richness of the three cities was not significant (*F* = 0.623, *P* > 0.05), the value of multisite beta-diversity (*β*_SOR_) was 0.260, and species spatial turnover processes seemed to be more important (*β*_NES_/*β*_SIM_ = 0.634). Table 3 shows that of the sites, pairwise beta-diversity between RC and XX was completely decided by spatial turnover (DISnes/sim = 0.000) and had the lowest dissimilarity value (*β*_sor_ = 0.167). Dissimilarity between RC and AX was mainly decided by nestedness (DISnes/sim = 2.429), while dissimilarity between XX and AX was decided by turnover and nestedness simultaneously (DISnes/sim = 1.143).

**Table 3:** Sorensen dissimilarities and the values among three counties.

City pairs^a^	DISsor	DISsim	DISnes	DISnes/sim	DIST(Km)^b^
RC∼XX	0.167	0.167	0.000	0.000	21.623
RC∼AX	0.190	0.056	0.135	2.429	15.086
XX∼AX	0.238	0.111	0.127	1.143	16.554

**Notes.**

a “RC”, “XX”, “AX” represent the three counties “Rongcheng County”, “Xiongxian”, and “Anxin County”, respectively.

b DIST means the linear distance between each city pair’s geometric centers.

### Avian-environment relationships along the urbanization gradient

Using Spearman correlation analysis, we obtained five environmental indexes that were significantly correlated with both breeding bird species richness and individual abundance. Substantial multiple collinearity existed among them (Table 4), and thus, PCA was conducted to extract the principle components. The ratio of the sample size (30) to the number of independent variables (five) analyzed was 6:1, which was fit for PCA; the Kaiser-Meyer-Olkin (KMO) value was 0.652, passing the Bartlett sphericity test (*P* < 0.001) (Zhang & Dong, 2004). Two principle components with eigenvalues >1 were extracted by PCA, PC 1 and PC 2, which contained 86.34% of the information of all the environmental variables to be separated. Based on the matrix in Table 5, we suggested that PC 1 is largely the reflection of tree species diversity and PC 2 represent for the effect of FHD. The expression of PC 1 is *y*_1_ = 0.269*Pedestrians + 0.522 ∗H′_tree + 0.386 ∗S_tree − 0.071 ∗C_tree − 0.238*FHD; and for PC 2 it is *y*_2_ = 0.071*Pedestrians − 0.274 ∗H′_tree − .042 ∗S_tree + 0.485 ∗C_tree + 0.640*FHD. Principle component regression produced two models, that is, *Y*_1_ = 1.543 + 0.206 ∗*y*_1_ + 0.347 ∗*y*_2_; *Y*_2_ = 3.025 + .0321 ∗*y*_1_ + 0.421 ∗*y*_2_. They described 60.9% and 54.3% of species richness and individual abundance, respectively (Table 6). After the transformation of coefficients, we got the final models, for breeding bird species richness it is ${\hat {Y}}_{1}=1.543+0.080\text{*Pedestrians}+0.012\mathrm{ \ast }{\mathrm{H}}^{{^{\prime}}}\text{_tree}+0.065\mathrm{ \ast }\mathrm{S}\text{_tree}+0.154\mathrm{ \ast }\mathrm{C}\text{_tree}+0.173\text{*FHD}$; for breeding bird individual abundance it is ${\hat {Y}}_{2}=3.025+0.116\text{*Pedestrians}+0.052\mathrm{ \ast }{\mathrm{H}}^{{^{\prime}}}\text{_tree}+0.106\mathrm{ \ast }\mathrm{S}\text{_tree}+0.181\mathrm{ \ast }\mathrm{C}\text{_tree}+0.193\text{*FHD}$. It should be noticed that all the variables in the above models have been log transformed.

**Table 4:** Multiple collinearity of the predicted factors.

	Pedestrians	H’_tree	S_tree	C_tree
H’_tree	.691^**^			
S_tree	.701^**^	.924^**^		
C_tree	.572^**^	.477^**^	.682^**^	
FHD	.474^**^	.190	.371^*^	.676^**^

**Notes.**

* represents *p* < 0.05.

** represents *p* < 0.01; for other descriptions, please see Table 1.

**Table 5:** PCA analysis results of correlated environmental factors.

	PC1	PC2
Eigenvalue	3.295	1.022
Relative percent variance (%)	48.926	37.410
Cumulative percent variance (%)	48.926	86.336
Pedestrians	.269	.071
H’_tree	.522	−.274
S_tree	.386	−.042
C_tree	−.071	.485
FHD	−.238	.640

**Table 6:** Regression analysis results between PCA scores and avian community indexes.

Targets	Predictors	Coefficient	T	Sig.	F	R^2^	adjR^2^
Species abundance	Constant	1.543	25.266	.000	21.047	.609	.580
	REGR factor score 2	.347	5.578	.000			
	REGR factor score 1	.206	3.313	.003			
Individual number	Constant	3.025	32.911	.000	16.012	.543	.509
	REGR factor score 2	.421	4.502	.000			
	REGR factor score 1	.321	3.429	.002			

## Discussion

Our results showed that a relatively poor avian community existed within the Xiong’an New Area. Although the landscape has experienced more than 2,000 years of agricultural exploitation, it still served as habitat for endangered species such as the raptors recorded in the field survey. Local avian biodiversity hotspots were distributed along the urban fringes, and significant differences existed along the urbanization gradient.

In the rural area, the typical wheat field-shelterbelt landscape seems hard for the persistence of small passerines; which probably is one of the major reasons why raptors were mostly recorded feeding on these birds in the urban fringes. Because of its large area, local special habitats could still sustain specific bird populations such as the Vinous-throated Parrotbill (*Paradoxornis webbianus*), and thus, the number of exclusive species recorded in the rural area was higher than that recorded in the urban area, which is consistent with the results of a previous study (Sengupta, Mondal & Basu, 2014). Within the urban area, densely constructed urban landscape mosaics with green patches acted as biodiversity hotspots (Enedino, Loures-Ribeiro & Santos, 2018; Fernandez-juricic & Jokimaki, 2001). However, for the three urbanized areas in this study, only two small parks existed within the urban area of XX. In RC and AX, there were only a few extremely fragmented green patches that acted as roads or courtyard greens. As a result, the avian community within the urban area of this study was in a poor state. Only urban fringes that connected urban and rural areas had some remaining secondary forests or plantations that were naturally occurring to some extent, with weeds, with shrubs and domestic garbage under the canopy that acted as an ideal constructed habitat for specific bird guilds (Evans et al., 2011; Evans, Newson & Gaston, 2009; Nielsen et al., 2014; Shwartz et al., 2013). Analysis results highlighted the importance of the urban fringe, which were consistent with previous studies (Geschke et al., 2018; Menon, Devi & Rangaswamy, 2016).

The statistical analysis results showed that there was a positive relationship between breeding bird community indexes and pedestrian number, while previous studies suggested a negative correlation (Morelli et al., 2018). Notably, both insectivores that favor naturally occurring food and omnivores that favor naturally and unnaturally occurring food peaked in the urban fringe. Combined distribution patterns suggested that avian food resources and ecosystem services (such as recreational services) coexisted in the narrow urban fringe, with birds and humans possibly gathering in the strip because they had no other choices. Interestingly, a gradient study conducted in Calcutta, India, with a similar field survey method suggested that a total of 37 avian species (26%) occur in all urbanization gradients, and in our study, there were nine species (24%). In the study in India, thirty-five species (26%) only existed in one specific gradient, 22% of which were recorded in sample points outside of the urban area, and 4% of the species were recorded in the urban area; in this study, 13 species existed in one gradient (35%), with 30% in sample points outside the urban area, and 5% of the species were recorded in the urban area (Pal et al., 2019). Based on our results, we suggested that similar studies in different regions will be valuable to check whether the urbanization gradient pattern had already been formed since the small cities at the Chinese county level.

At the regional scale, beta-diversity combined with species richness acted as the decisive index for species diversity (Harrison, Ross & Lawton, 1992). For biodiversity conservation and landscape planning of reserves, beta-diversity has typically been adopted as the key ecological index for regional master plans (Socolar et al., 2016), while the decomposition of beta-diversity could provide further references for conservation biology (Si, 2014). Although previous studies have suggested that spatially independent large parks could favor beta-diversity through species turnover (Quinn & Harrison, 1988; Wiersma & Urban, 2005), the dispersal limitation of some small passerines and insects that act as their food resources, combined with probable cascade effects that could lead to regional extinction of some bird species (Bauer & Hoye, 2014; Pearson, 2006). In comparison to nestedness, the spatial turnover process contributed more to total beta-diversity, suggesting that bird habitats within all three cities are valuable to protect (Angeler, 2013). Dissimilarity between RC and XX was driven by spatial turnover, corresponding with the largest distance between city pairs between them, so we speculated that spatial isolation probably plays a key role in the formation of avian communities within them (Russell et al., 2006). Conversely, pairwise dissimilarity between RC and AX was mainly determined by nestedness, corresponding with the minimum green space area within AX (Xu et al., 2017), while related studies have suggested that avian communities within small green patches are usually a subset of nearby large patches (Villegas Vallejos, Padial & Simoes Vitule, 2016; Wang et al., 2013). Pairwise dissimilarity between XX and Anxin was simultaneously determined by spatial turnover and nestedness, and the possible mechanism could be the mixed effects of relatively short city pair distance and the separation of large waterbodies (Baiyangdian Wetland). Based on these results, we suggest that spatial isolation probably plays a key role in the formation of avian communities within the three cities.

For the avian-environment relationship, our results showed that species diversity of trees and FHD are important environmental indexes for breeding birds. Abundant tree species and substantial maturity of vegetation vertical structure could supply food and habitats for more bird guilds (Canedoli, Manenti & Padoa-Schioppa, 2018). Some studies also have found a positive influence of canopy cover (Schuetz & Schulze, 2015; Threlfall et al., 2017), although shrub cover seems to be more important for both the alpha- (Newman et al., 2018) and beta-diversities (Munoz-Pedreros et al., 2018) of bird species. The deficiency in shrub cover within our study area was probably one of the major reasons for the poor state of the total avian community. While tree species diversity and FHD are key environmental factors for species richness and individual abundance, we propose that these two habitat indexes are critical for both the occurrence and occupancy of breeding birds.

One of the most important contributions of this study is the quantification and localization of bird diversity hotspots in Xiong’an New Area, and combined with the difference among the avian communities of the three constructed areas, decision support could be provided for the planning of key nodes of ecological corridors. Based on our results, we suggest that planners and decision makers pay more attention to biodiversity conservation and establish new urban green spaces in the three cities guided by avian biodiversity conservation while considering increasing vegetation structure complexity to promote avian species alpha-diversity within habitat patches. On the other hand, based on the relatively high total beta-diversity value, we recommend that different bird habitats within each city be constructed based on current conditions to promote alpha-diversity of the avian community within each city. Based on the dissimilarity and the drivers of the avian communities of city pairs, we advise that habitats within XX and RC that sustain more avian species receive more attention.

## Conclusions

To conclude, significant differences of avian community configuration occurred in urbanization gradients of our research area, urban fringes contained all peaks of species richness and individual abundance of different species guilds; significant differences of bird species composition also occurred among the three constructed areas of this study.

Dissimilarity indexes of avian communities within city pairs could provide useful suggestions for regional master plans. The significant influences of tree species diversity and FHD for both the occurrence and occupancy of breeding birds highlighted the value of protecting the existing mature plantations in the urban fringes, as well as the construction of new plantations that are both species abundant horizontally and structure mature vertically.

##  Supplemental Information

10.7717/peerj.7961/supp-1 Appendix S1 Appendix 1: Bird species recorded in the field survey and their residence and feeding types a. Protection level refer to *List of Endangered and Protected Species of China* (Ministry of Forestry of the PRC, 1989).

10.7717/peerj.7961/supp-2 Data S1 Breeding bird species richness and individual density on urbanization gradient and the corresponding environment variables

## Additional Information and Declarations

### Field Study Permissions

The following information was supplied relating to field study approvals (i.e., approving body and any reference numbers): Field experiments were approved by the Research Council of Research Center for Eco-environmental Science, Chinese Academy of Sciences.

### Data Availability

The following information was supplied regarding data availability: The raw measurements are available in the Supplemental Files.
